# Multiple electrolyte imbalances and mixed acid-base disorder posing a diagnostic dilemma: a case report

**DOI:** 10.1186/s13256-019-2330-2

**Published:** 2020-01-20

**Authors:** Fortune O. Alabi, Christopher O. Alabi, Rafaela G. Basso, Nadia Lakhdar, Adebanke O. Oderinde

**Affiliations:** 1 Florida Lung Asthma and Sleep Specialists, Orlando, FL USA; 2 St. Matthew’s University School of Medicine, Orlando, FL USA

**Keywords:** Alcohol intoxication, Lactic acidosis, Mixed acid-base disorder, Electrolyte imbalance, Hypokalemia, Hypomagnesemia, Hypocalcemia, Hypophosphatemia, Lab, History

## Abstract

**Background:**

In clinical practice, both the history and laboratory testing are paramount to making an accurate diagnosis. Situations in which laboratory findings and patient history are not congruent pose a diagnostic dilemma. We report a case of a young woman presenting with a myriad of electrolyte and acid-base disorders. Difficulty in reaching a unifying diagnosis persisted due to discordant patient history. We believe this case shows that lab findings will clearly portray the problems a patient has and should be given more credence in a case where the history is discordant with lab findings.

**Case presentation:**

A 28-year-old Hispanic American woman presented to the emergency room of our institution with a complaint of painless and sudden onset of stiffness in her upper and lower limbs. Associated weakness worse in the distal limbs was also reported. She experienced shortness of breath with minimal exertion, diaphoresis, and anxiety. Her vital signs revealed tachycardia without corresponding fever. She was conscious, oriented, and alert. Her physical exam revealed dry mucous membranes and warm extremities. She denied recent consumption of a large carbohydrate meal, diarrhea, vomiting, use of laxatives, and use of alcohol or recreational drugs. She vaguely described two previous similar episodes in the last 7 months that spontaneously resolved. Her medical history was significant only for hypothyroidism treated with daily levothyroxine tablets. Laboratory analysis revealed the following abnormalities: an elevated anion gap with significant lactate, hypokalemia, hypomagnesemia, elevated mean corpuscular volume, elevated mean cell hemoglobin, and elevated liver enzymes with aspartate aminotransferase/alanine aminotransferase ratio > 2. She was hydrated with balanced crystalloids, and her electrolyte deficiencies corrected. The etiology of her multiple electrolyte abnormalities was unclear because alcohol use was vehemently denied. Extensive evaluation for causes of electrolyte disorder was undertaken, which was unrevealing. On further interrogation, she admitted to recent alcohol intoxication and several episodes of vomiting before presentation. She was advised to refrain from alcohol use and discharged afterward.

**Conclusion:**

Both patient history and laboratory analysis have a role in identifying and confirming a diagnosis. In cases in which laboratory tests are incongruous with reported history, making a unifying diagnosis can be challenging or delayed. The importance of taking a comprehensive history cannot be overemphasized, but history provided by patients may be prone to intentional or unintentional distortion, whereas laboratory findings are more objective. The case presented underscores why the lab findings should be given credence in cases in which there is discordance between lab results and the provided patient history.

## Background

In order to accurately diagnose any condition a patient presents with, a well-taken history is required. A detailed patient history characterizes symptoms and provides insight into risk factors, and both are essential for obtaining a correct diagnosis. Blood test results play a vital role in confirming the diagnosis; however, in most cases, the correct diagnoses are made on the basis of a well-taken history [1]. In the case presented, we share the diagnostic dilemma we encountered when the results of laboratory-performed blood tests were incongruous with a patient’s provided history. We believe this case shows that lab findings will clearly portray the problems a patient has and should be given more credence in a case where the history is discordant with lab findings.

## Case presentation

A 28-year-old stay-at-home Hispanic American wife visiting from out of state was brought to the emergency room of our institution by her relatives on account of sudden-onset stiffness and weakness of both her upper and lower extremities. The symptoms affected her wrists, ankles, fingers, and toes and began acutely while waiting in line in direct sunlight for a theme park ride. She also reported her heart beating faster than normal, shortness of breath with minimal exertion, diaphoresis, anxiety, numbness, and loss of mobility in her right leg. She denied fever, headache, and musculoskeletal pain. On more detailed questioning, she denied consuming a large carbohydrate meal and had no episodes of diarrhea, vomiting, use of laxatives, licorice, alcohol use, smoking, or recreational drugs. She denied exposure to industrial chemicals and lives in a recently constructed building. The patient reported consuming a regular diet with no recent dietary modifications. She has a history of hypothyroidism caused by Hashimoto’s thyroiditis since age 10 treated with 88 μg of levothyroxine daily, but she has been otherwise healthy all her life. She claimed to have experienced similar symptoms for the first time approximately 7 months prior. At that time, only her upper extremities were involved, and symptoms occurred during a traffic stop. A similar episode occurred 2 weeks prior to presentation with acute onset, lasting 2 hours with resolution after rest and hydration. She had no family history of similar symptoms, and a review of her systems yielded no additional information.

Examination revealed a well-groomed young woman whose vital signs showed tachycardia of 150 beats/minute, blood pressure 133/89 mmHg, and body temperature 98.4 °F. She was alert and oriented to person, place, time, and situation. No focal neurological deficits were observed, and she had preserved sensation with normal speech and coordination. Of note, she had dry mucous membranes. The rest of her physical examination yielded no additional information.

Admission blood testing revealed hypokalemia (K^+^ level 2.3 mmol/L), hypomagnesemia (Mg^+^ level 1.2 mg/dl), hypochloremia (Cl^−^ level 81 mg/dl), normal calcium (Ca^+^ level 8.8 mg/dl), normal sodium (Na^+^ level 139 mmol/L), severely elevated lactic acid (12.93 mmol/L), anion gap of 31 mmol/L, partial pressure of carbon dioxide 36.6 mmHg, HCO_3_^−^ 27.3 mmol/L, and pH 7.48. She had features of acute kidney injury with glomerular filtration rate 68 ml/minute and creatinine 1.1 mg/dl (her normal baseline is about 0.5 mg/dl), high thyroid-stimulating hormone 17 U/L, normal T4 at 1.25 ng/dl, alanine aminotransferase (ALT) 53 U/L, aspartate aminotransferase (AST) 113 U/L, serum ethanol < 10 mg/dl, macrocytosis with mean corpuscular volume 107.1 fl/cell, mean corpuscular hemoglobin 38 pg/cell, and red blood cell distribution width 17.7. Her other metabolic panel results were essentially within normal limits, including negative troponin and creatinine kinase. An electrocardiogram obtained in the emergency department showed sinus tachycardia, and her urine, which was hazy, had 3+ bacteria and 3+ leukocyte esterase. Her complete blood count revealed no leukocytosis. Her macrocytosis was thought to be due to her hypothyroidism, and her deranged liver enzymes with an AST/ALT ratio > 2 suggested alcoholic liver injury, but she vehemently denied alcohol consumption. Other possibilities, such as rhabdomyolysis and hemolysis, were entertained but quickly ruled out. Her serum pH was noted to be alkalemic despite her severely elevated anion gap, indicating a mixed acid-base disorder, but the cause could not be explained.

She received an adenosine injection in the emergency room for possible supraventricular tachycardia, boluses of crystalloid, and replacement of potassium and magnesium. Repeat serum electrolytes after 4 hours showed corrected magnesium of 2 mg/dl, but surprisingly, her low potassium further reduced to 1.8 mmol/L despite receiving supplementation. Her serum calcium level was reduced to 7.3 mg/dl from the previous normal level of 8.8 mg/dl, and her phosphorus level was reduced to 2.2 mg/dl at this time from a previously normal level of 3.4 mg/dl. She was referred to the intensive care unit on account of severe hypokalemia, where she was monitored on telemetry while electrolyte replacement protocols were carried out. She was started empirically on 1-g ceftriaxone intravenously secondary to abnormal urinalysis showing pyuria with 3+ bacteria, which, in the presence of lactic acidosis, brought up the possibility of sepsis.

After 28 hours of admission, her serum electrolytes normalized. The cause of all her symptoms and electrolyte derangement was still unknown. Other laboratory findings included elevated indirect bilirubin of 1.7 mg/dl, direct bilirubin of 2.1 mg/dl, mildly elevated prothrombin time 15.8 seconds, international normalized ratio 1.3, mildly reduced albumin of 3.1 g/dl, mildly reduced total protein of 5.8 g/dl, low folate at 3.9 ng/ml, and normal vitamin B_12_ of 556 pg/ml. The patient was asked about previous alcohol use or abuse based on elevated transaminases in a pattern suggestive of alcoholic liver disease. She vehemently denied alcohol use until eventually admitting consumption of excessive amounts of alcohol when she was questioned in the absence of her relatives. Her gamma-glutamyl transferase level was elevated at 380 U/L, which also supports alcoholic liver disease. She admitted alcohol intoxication with “hangover” symptoms 2 days prior to presentation. She also admitted to ten episodes of nonbloody, nonbilious emesis the day before presentation. A diagnosis of electrolyte disorders with lactic acidosis secondary to alcohol intake was consequently made. She was discharged after another 11 hours and was cautioned to refrain from alcohol use. The antibiotic was discontinued prior to discharge because she was asymptomatic.

## Discussion

The management of a 28-year-old woman who presented with acute-onset muscular stiffness, a history of mixed acid-base imbalance, and multiple electrolyte derangement brought a number of differential diagnoses to mind. She denied use of alcohol in her initial history several times, and as a result, we needed to try to explain what other conditions could cause her symptoms.

Some of the differential diagnoses considered included hypothyroid myopathy because of her history of hypothyroidism, but this was easily ruled out because of her normal free T4. On account of her urinalysis showing pyuria, bacteriuria, and elevated leukocyte esterase with lactic academia, a diagnosis of sepsis was considered. The absence of fever, leukocytosis, and the severe electrolyte derangements made sepsis unlikely. The abnormal urinalysis was ascribed to asymptomatic bacteriuria. We also considered thyrotoxic periodic paralysis (TPP) because she was taking thyroid supplement medication, and excessive exogenous intake of levothyroxine can cause TPP, although it is commonly seen in endogenous cases of thyrotoxicosis such as Graves disease [2]. TPP was discounted easily because of normal free T4 and the acid-base disorder, which could not be explained by this entity. Hypokalemic periodic paralysis was also considered but ruled out because it could not explain the other electrolyte imbalances and lactic acidosis. Alcohol abuse remained the best rationale for her constellation of symptoms and provided plausible mechanisms for both the electrolyte disorders and acid-base disorders.

Alcohol abuse has been linked to a variety of abnormalities such as acid-base disorders, dehydration, and electrolyte imbalances [3]. Metabolic acidosis with anion gap, respiratory alkalosis, metabolic alkalosis, and mixed disturbances can be seen in patients who abuse alcohol, and the presence of each varies from patient to patient [4–6].

The production of lactic acid and ketone bodies (β-hydroxybutyric acid and acetoacetate) is the cause of acidosis in patients who abuse alcohol. The lactic acidosis is due to elevated hepatic nicotinamide adenine dinucleotide + hydrogen (NADH)/reduced nicotinamide adenine dinucleotide (NAD^+^) ratio that occurs with alcohol metabolism.

**Figure:**
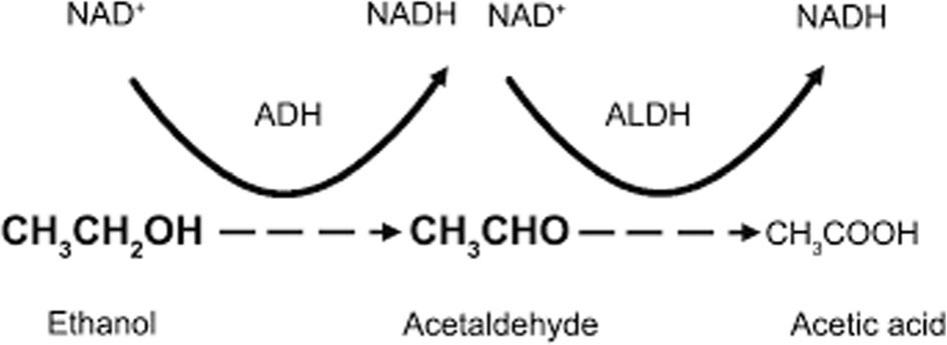

The excess NADH favors lactate production from pyruvate.

**Figure:**
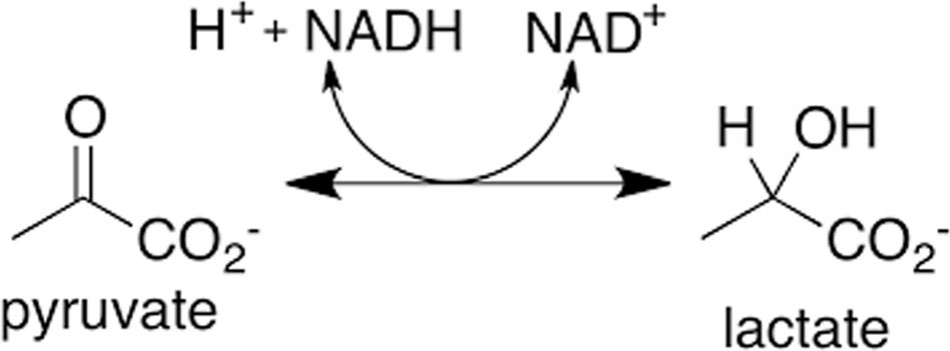

As a result, less pyruvate will be available for gluconeogenesis [7]. Furthermore, low NAD^+^ will inhibit the Krebs cycle because three enzymes (isocitrate dehydrogenase, α-ketoglutarate dehydrogenase, and malate dehydrogenase) in the pathway require NAD^+^ for their reduction reactions. Severe forms of lactic acidosis resulting from alcohol abuse are seen when the patient has coexisting thiamine deficiency and tissue hypoperfusion, such as in sepsis, hypovolemia, or heart failure. Thiamine deficiency inhibits the formation of acetyl coenzyme A (CoA) from pyruvate because the oxidation of pyruvate to acetyl CoA by pyruvate dehydrogenase requires thiamine as cofactor. Thiamine deficiency inadvertently favors formation of more lactic acid from pyruvate when NAD^+^ level is low. We believe the cause of severe lactic acidosis in our patient was due to the combination of alcohol-induced lactic acidosis and systemic hypoperfusion due to intravascular volume depletion. Hepatic NADH/NAD^+^ ratio usually returns toward the normal baseline once ethanol consumption ceases, allowing all the impeded pathways to return to normal [8].

Acetic acid, one of the oxidative products of alcohol, becomes converted to acetyl CoA in the liver and from there becomes a substrate for fatty acid synthesis or formation of ketones such as acetoacetate, β-hydroxybutyric acid, or acetone. This is the cause of a mild or moderate ketone production initially seen after recent alcohol ingestion [6, 9].

**Figure:**
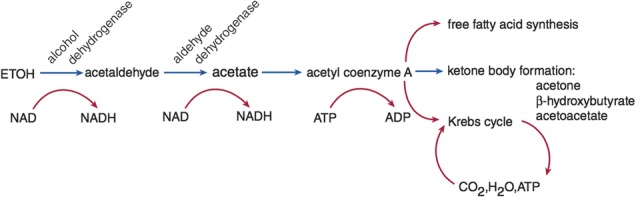

Severe ketoacidosis usually occurs when blood alcohol levels begin to reduce, and decreased oral food intake causes reduced insulin release, which triggers an increase in counterregulatory hormones glucagon, norepinephrine, and cortisol, resulting in the formation of more ketone bodies from the broken-down fatty acids [10–13]. An elevated NADH/NAD^+^ ratio associated with alcohol intoxication further encourages the conversion of acetoacetate to β-hydroxybutyrate, making β-hydroxybutyrate the predominant ketoacid in alcoholic ketoacidosis [14].

Alkalosis in patients with alcohol abuse may be due to loss of hydrogen chloride as a result of protracted vomiting or respiratory alkalosis associated with alcohol withdrawal [15]. The respiratory alkalosis tends to be more significant in patients with sepsis, pain, or cirrhosis because of hyperventilation. A mixed acid-base disturbance is seen when the alkalosis coexists with acidemia from the aforementioned mechanisms. Our patient presented with an elevated anion gap of 31 mmol/L and had a blood pH of 7.48, which is alkalemic and fits the description. According to a study, about 78% of patients admitted with acid-base disorders related to alcohol present with mixed disturbances [9]. An increased anion gap greater than the decrease in bicarbonate concentration will be seen when this occurs, just as in our patient.

The hypokalemia seen in our patient could have been caused by gastrointestinal loss from vomiting. Aldosterone release in response to hypovolemia leads to increased kaliuresis and can be more severe with associated hypomagnesemia, as in our patient. A normal magnesium level is required to block the renal intracellular renal outer medullary potassium (ROMK) channels, which are located on the apical membrane of the distal nephron and limit outward potassium secretion from the distal tubular cells. Hypomagnesemia reduces intracellular magnesium, thereby affecting the inhibition of the ROMK channels and causing urinary potassium wasting, which worsens hypokalemia [16, 17]. Hypokalemia with concomitant hypomagnesemia as described above will be refractory to correction until the magnesium is corrected [18].

Hypomagnesemia in our patient can be explained by the direct magnesiuric effect of alcohol consumption [19, 20] or alcohol-induced renal tubular dysfunction, which causes urinary loss of magnesium [16]. Intracellular magnesium shift can also cause hypomagnesemia, possibly brought on in our patient when acidosis was being corrected in the hospital with fluids and by the release of insulin in response to glucose-containing fluids. Elisaf *et al.* found hypomagnesemia in about 30% of patients with chronic alcohol abuse in their cohort study, and it should be noted that magnesium levels help regulate levels of other electrolytes, such as phosphate, calcium, and potassium, and parathyroid hormone resistance can be seen in hypomagnesemia [21, 22].

Hypophosphatemia in our patient may be explained by ethanol directly causing structural changes in the phospholipid bilayer of the membrane of renal tubular cells and generalized tubular dysfunction, which decreases renal threshold for phosphate excretion. The tubular dysfunction usually improves within days of abstinence from alcohol [23]. Stimulation of phosphofructokinase by a rise in body pH resulting from excessive vomiting or respiratory alkalosis (from alcohol withdrawal) can also cause reduction in phosphate levels because it is used as a substrate in glycolysis. Additionally, intravenous dextrose fluid, which stimulates insulin release, promotes phosphate uptake by the cells as phosphorylated glucose intermediates [24].

Hypocalcemia in our patient could have been due to direct effect of alcohol, which causes urinary calcium loss [25]. Alcohol also decreases the activity of Na^+^,K^+^-ATPase in the renal proximal convoluted tubule cells, causing a decrease in the tubular reabsorption of calcium, which could have been worsened by concomitant hypomagnesemia [26, 27]. Another proposed mechanism that links hypocalcemia to hypomagnesemia is the peripheral resistance of parathyroid hormone secondary to hypomagnesemia, which prevents normal physiologic maintenance of normal serum calcium level [22]. Also, alcohol has a direct effect in reducing vitamin D metabolism, which results in decreased intestinal calcium absorption [28]. Even though our patient’s serum vitamin D level was not checked in the course of this admission, the possibility of a low vitamin D level cannot be ruled out. At 1 month after discharge, our patient remained symptom-free.

## Conclusion

Hampton *et al*. reported that “a careful history will lead to the diagnosis 80% of the time” [1], but our patient’s case is an example of a time when a carefully taken history may hinder determining the right diagnosis in a timely manner. The laboratory results in our patient clearly pointed to alcohol abuse. In order to prevent the cognitive biases of anchoring and premature closure, we decided to give the patient the benefit of the doubt and diligently explored other diagnostic possibilities based on her claim that she did not drink alcohol. This is a case where believing the laboratory results may have saved resources and prevented an unnecessary delay in diagnosis.

## Data Availability

Available on demand.

## Abbreviations

ALT: Alanine aminotransferase
AST: Aspartate aminotransferase
CoA: Coenzyme A
NAD^+^: Reduced nicotinamide adenine dinucleotide
NADH: Nicotinamide adenine dinucleotide + hydrogen
ROMK: Renal outer medullary potassium channel
TPP: Thyrotoxic periodic paralysis
